# Complete chloroplast genome and phylogenetic analysis of *Lonicera caerulea* var. *edulis* (Caprifoliaceae)

**DOI:** 10.1080/23802359.2023.2180309

**Published:** 2023-02-24

**Authors:** Chenqiao Zhu, Xinyu Sun, Qiang Fu, Ying Zhan, Songlin Li, Yu Liu, Min Yu, Dong Qin, Lijun Zhang, Junwei Huo

**Affiliations:** a College of Horticulture & Landscape Architecture, Northeast Agricultural University, Harbin, China; b National-Local Joint Engineering Research Center for Development and Utilization of Small Fruits in Cold Regions, National Development and Reform Commission, Harbin, China; c Key Laboratory of Biology and Genetic Improvement of Horticultural Crops (Northeast Region), Ministry of Agriculture and Rural Affairs, Harbin, China; d College of Life Science, Northeast Agricultural University, Harbin, China; e Heilongjiang Institute of Green Food Science, Harbin, China

**Keywords:** Blue honeysuckle, Haskap

## Abstract

*Lonicera caerulea* var. *edulis*, known as “blue honeysuckle” or “Haskap,” is a deciduous shrub that belongs to the Caprifoliaceae family. Characterized by the high cold hardiness and high quality of fruit, it has become a novel cash crop in cold regions worldwide. The lack of available chloroplast (cp) genome information limits studies of its molecular breeding and phylogeny. Here, the complete cp genome of *Lonicera caerulea* var. *edulis* was assembled and characterized for the first time. It was 155,142 bp in length with 38.43% of GC content, including 23,841 bp inverted repeat regions (IRs), an 88,737 bp large single-copy region (LSC), and an 18,723 bp small single-copy region (SSC). A total of 132 genes, including 85 protein-coding genes, 8 rRNA genes, and 39 tRNA genes were annotated. Phylogenetic analysis indicated that *L. caerulea* var. *edulis* was closely related to *L. tangutica*. These data and results provide a valuable resource for the development of breeding tools and genetic diversity studies for *L. caerulea*.

## Introduction

*Lonicera caerulea* var. *edulis* (Linn.) Turcz. ex Herd. 1864 (blue honeysuckle) is a deciduous shrub that produces blue-black edible berries with a high concentration of anthocyanins, phenolic acids, and polyphenols (Becker and Szakiel 2019). As a circumpolar species, its tree and flower are hardy to −50 °C and −7 °C, respectively (Plekhanova 2000, Hummer et al. 2012). Thus, its cultivation and breeding have undergone fast progress during the past two decades in the cold regions of the Northern Hemisphere, especially in Russia, China, and Canada (Huo et al. 2005, Gerbrandt et al. 2017).

Despite its importance, the phylogenetic position of *L. caerulea* in *Lonicera* is still less clear. It was designated as the only species of subsection *Caeruleae* in section *Isika* of the subgenus *Chamaecerasus* (Rehder 1903, Rehder 1913, Hara 1983, Wu and Hong 2011). Because of the lack of plant material or sequence data of *L. caerulea*, whether *L. caerulea* should be classified under *Isika* is still controversial, and the phylogenetic relationship between subsection *Caeruleae* and other subsections in section *Isika* remains unclear (Theis et al. 2008, Nakaji et al. 2015, Liu et al. 2018). Due to the genomic characteristics of nonrecombination, high conservation, and uniparental inheritance, the complete chloroplast (cp) genome has been accepted as a powerful tool for phylogenetic studies and molecular breeding (Bi et al. 2018). To date (August 2022), more than 40 versions of *Lonicera* cp assemblies have been released in GenBank, but *L. caerulea* has not been included.

Here, the cp genome of *L. caerulea* var. *edulis* was assembled and characterized for the first time and a plastome phylogeny of the genus *Lonicera* was reconstructed, providing useful genetic resources for the molecular breeding of *L. caerulea* and new insight into the phylogeny of *Lonicera* species.

## Materials

Fresh leaves were obtained from a plant of *L. caerulea* var. *edulis* cultivated at the Horticulture Experimental Station (126.73 N, 45.74E) of Northeast Agricultural University (NEAU), Harbin, China. “LE02” was initially collected as a twig from the Lesser Khingan Mountains by Junwei Huo (junweihuo@aliyun.com) in 2002 and was propagated by cutting. The botanical identification of “LE02” (Gui and Hu 2002, Zhang 2012) was accomplished by Professor Xiuju Wu (xiujuwu@neau.edu.cn). A specimen was deposited at NEAU (Junwei Huo, junweihuo@aliyun.com) under the voucher number “LE-02”.

## Methods

Total genomic DNA was extracted from 0.1 g of fresh leaves using a modified CTAB method (Doyle and Doyle 1986). A paired-end library with an average insert size of 350 bp was constructed using the MGIEasy PCR-Free DNA Library Prep Set and sequenced on the DNBSEQ™-T7 platform (MGI Tech Co., Ltd., Shenzhen, China). The raw reads were filtered using SOAPnuke (Chen et al. 2017) with the parameters “-lowQual = 20, -nRate = 0.005, qualRate = 0.5” (Supplementary File 1 and 2). The cp genome was assembled using GetOrganelle (Jin et al. 2020) with a k-mer set of “21, 45, 65, 85, 105” (Supplementary File 3 and Figure S1). The assembled genome was annotated using GeSeq (Tillich et al. 2017) and CPGView (Liu et al. 2022a). The 24 complete *Lonicera* cp genomes sequences were selected for phylogenetic analysis according to recent studies (Figure 3). The 8 representative species in Caprifoliaceae and Adoxaceae were selected according to classical taxonomy (Wu and Hong 2011, The Angiosperm Phylogeny Group et al. 2016, Stevens 2017) and recent molecular studies (Theis et al. 2008, Nakaji et al. 2015, Bai et al. 2017, Liu et al. 2018, Wang et al. 2020, Wang et al. 2021). All of the sequences were aligned using MAFFT v7.505 (Katoh and Standley 2013) and then trimmed using trimAL (Capella-GUTIéRREZ et al. 2009) in the “-automated1” pattern. The maximum likelihood tree was constructed using IQ-TREE 2.2.0.3 with the best-fit model of “TVM + F + I + I+R6” and 1,000 ultrafast bootstraps (UFBoot) replicates for branch support (Minh et al. 2020).

## Results and discussion

**Figure 1.**
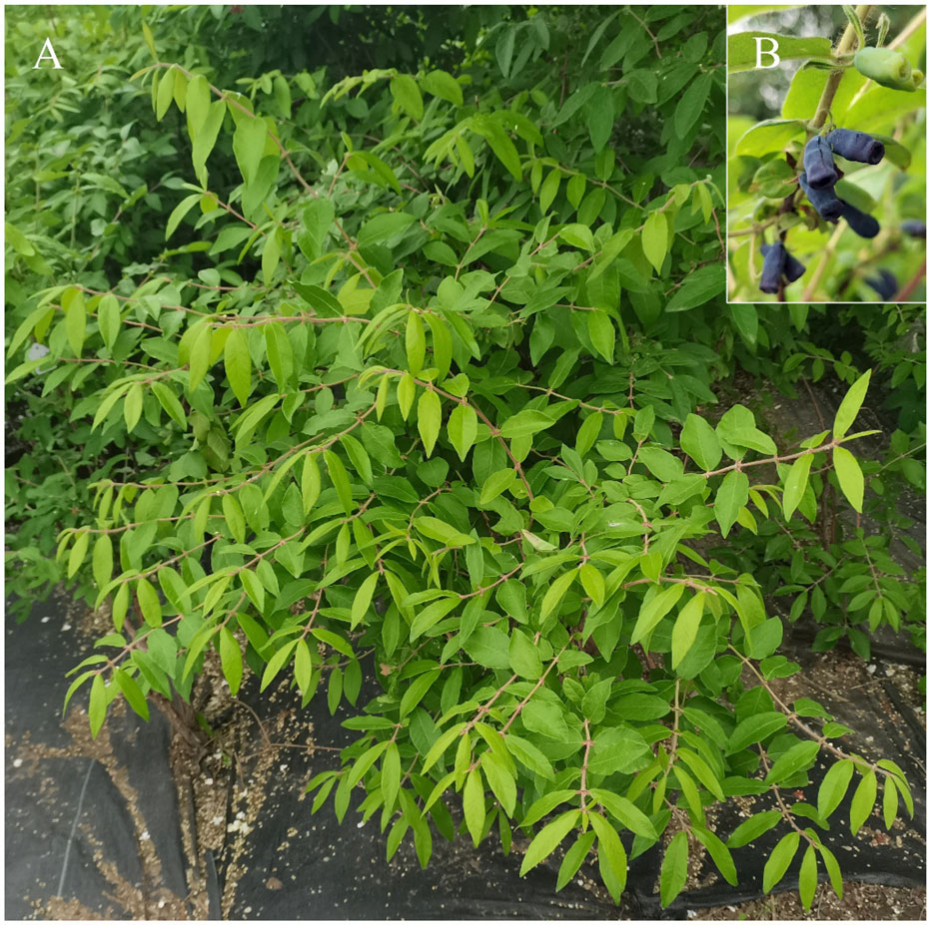
Tree (A) and fruit (B) morphologies of *Lonicera caerulea* var. *edulis*. Note: location, Horticulture Experimental Station (126.73N, 45.74E), Northeast Agricultural University (NEAU), Harbin, China; botanical identification, Pro. Xiuju Wu; photograph, Chenqiao Zhu.

*L. caerulea* var. *edulis* (Figure 1) is one of the three diploid varieties (2n = 2x = 18) of *L. caerulea* and is a native species in northeastern Asia (Plekhanova 2000). Due to its widespread wild distribution and primitive karyotype, it is not only considered an ancestor of tetraploid *L. caerulea* but also has the potential to be an excellent material for ploidy breeding and a model system for gene function studies (Huo et al. 2005).

**Figure 2.**
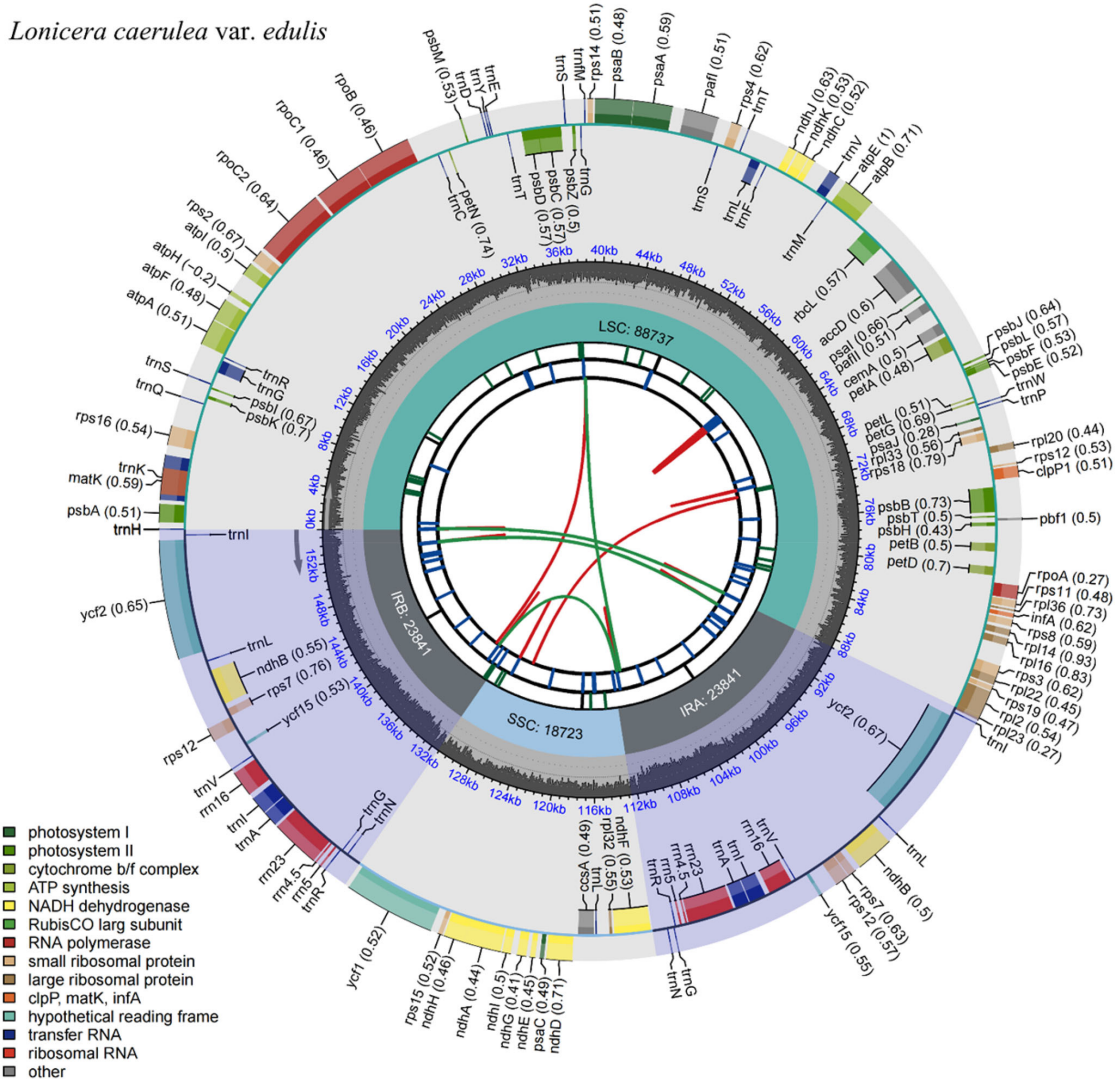
Feature map of *Lonicera caerulea* var. *edulis* chloroplast genome. From the center outward, the first track shows the dispersed repeats, which consist of direct (red arc) and Palindromic (green arc). The second track shows the long tandem repeats as short blue bars. The third track shows the short tandem repeats or microsatellite sequences as short bars with different colors (Black: complex repeat; Green: 1-unit repeat). The small single-copy (SSC), inverted repeat (IRa and IRb), and large single-copy (LSC) regions are shown on the fourth track. The GC content is plotted on the fifth track. The annotated genes are shown on the sixth track (codon usage bias is displayed in the parenthesis) and their functional classifications are color-coded in the bottom left corner. The transcription directions for the inner and outer genes are clockwise and anticlockwise, respectively.

The complete cp genome of *L. caerulea* var. *edulis* is 155,142 bp in length, with 38.43% of GC content. It includes a pair of IRs of 23,841 bp, which separate the LSC of 88,737 bp from the SSC of 18,723 bp (Figure 2). A total of 132 genes were annotated, including 85 protein-coding genes (PCGs), 8 rRNA genes (rRNAs), and 39 tRNA genes (tRNAs). Among them, 5 PCGs and 8 tRNAs were repeated in IRs, ten PCGs and 7 tRNAs contained one intron, *pafI* contained two introns, and *rps12* underwent trans-splicing (Table 1, Figure S2 and S3).

**Figure 3.**
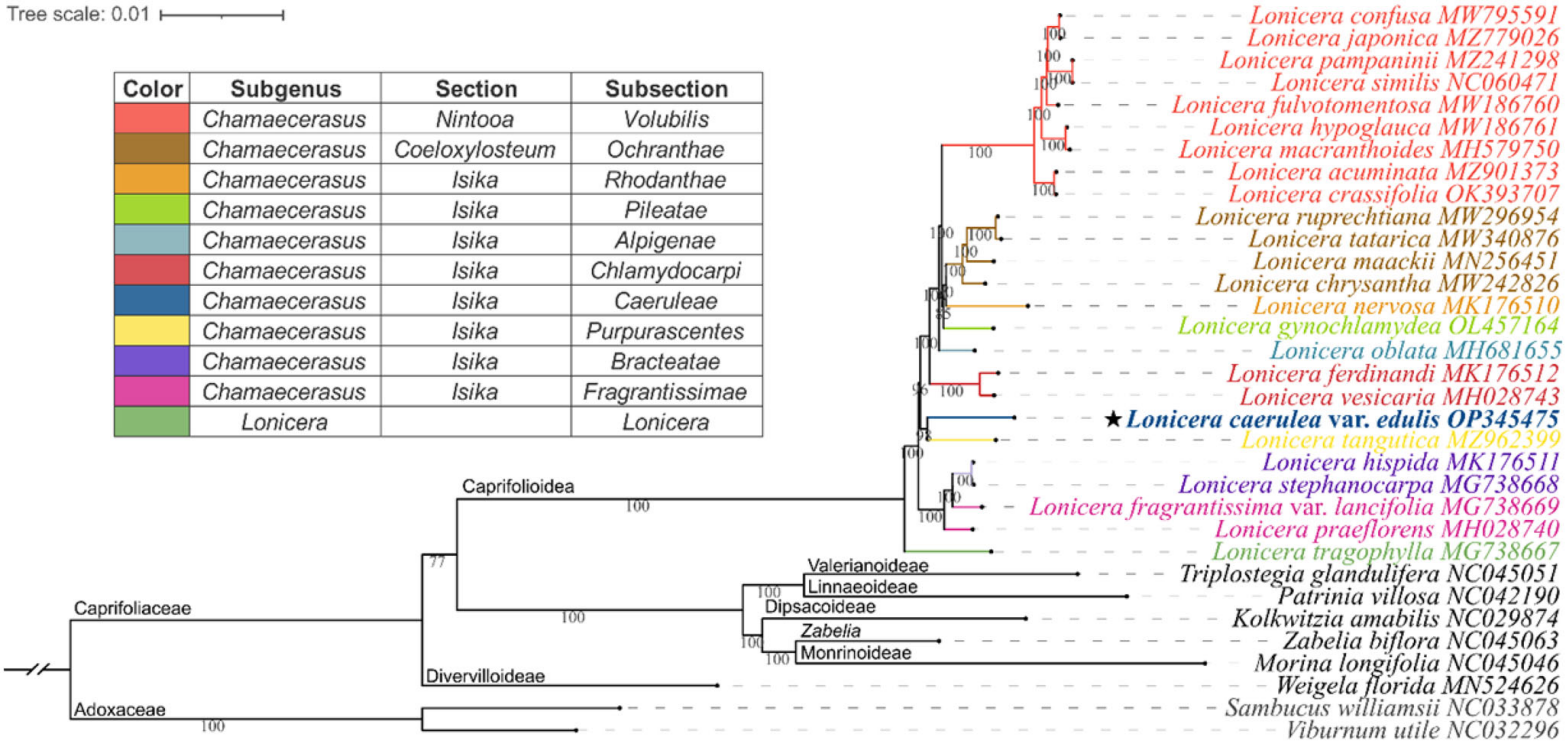
**Phylogenetic position of *Lonicera caerulea* var. *edulis* inferred from maximum likelihood (ML) based on 33 complete chloroplast genomes**. The subclassification of the *Lonicera* species examined in this study is according to Rehder (1903, 1913), and the representative colors of the subsections are shown on the left diagram. The subfamily classification was labeled according to Wang et al. (2020). The Numbers shown next to the nodes are bootstrap support values based on 1,000 replicates. The following sequences were used: MW795591 (Liu et al. 2022b), MZ779026 (Zhang et al. 2022), MZ241298 (Jiang et al. 2021), NC060471 (Wei et al. 2021), MW186760 (Yu et al. 2021), MW186761 (Gu et al. 2021), MH579750 (Hu et al. 2018), MZ901373 (Yang et al. 2022), OK393707 (Chen et al. 2022), MW296954 (Gu et al. 2022), MW340876 (Yuan et al. 2021), MN256451 (Jia et al. 2020), MW242826, MK176510 (Liu et al. 2018), OL457164 (Mo et al. 2022), MH681655 (Zhu et al. 2019), MK176512 (Liu et al. 2018), MH028743 (Kang et al. 2018), OP345475 (assembled in the present study), MZ962399 (Wang et al. 2022), MK176511 (Liu et al. 2018), MG738668 (Fan et al. 2018), MG738669 (Fan et al. 2018), MH028740 (Kang et al. 2018), MG738667 (Fan et al. 2018), NC045051 (Wang et al. 2020), NC042190, NC029874 (Bai et al. 2017), NC045063 (Wang et al. 2020), NC045046 (Wang et al. 2020), MN524626 (Wang et al. 2020), NC033878 (Fan et al. 2018), NC032296 (Huang and Cronk 2015).

**Table 1.** Gene annotation summary of *Lonicera caerulea* var. *edulis* chloroplast genome.

Gene category	Number	Name
Protein-coding genes	85	*ycf2* (×2), *ndhB* (×2) (i), *rps7* (×2), *rps12* (×2) (i), *ycf15* (×2), *psbA*, *matK*, *rps16* (i), *psbK*, *psbI*, *atpA*, *atpF* (i), *atpH*, *atpI*, *rps2*, *rpoC2*, *rpoC1* (i), *rpoB*, *petN*, *psbM*, *psbD*, *psbC*, *psbZ*, *rps14*, *psaB*, *psaA*, *pafI* (ii), *rps4*, *ndhJ*, *ndhK*, *ndhC*, *atpE*, *atpB*, *rbcL*, *accD* (i), *psaI*, *pafII*, *cemA*, *petA*, *psbJ*, *psbL*, *psbF*, *psbE*, *petL*, *petG*, *psaJ*, *rpl33*, *rps18*, *rpl20*, *clpP1*, *psbB*, *psbT*, *pbf1*, *psbH*, *petB*, *petD*, *rpoA*, *rps11*, *rpl36*, *infA*, *rps8*, *rpl14*, *rpl16*, *rps3*, *rpl22*, *rps19*, *rpl2* (i), *rpl23*, *ndhF*, *rpl32*, *ccsA*, *ndhD*, *psaC*, *ndhE*, *ndhG*, *ndhI*, *ndhA* (i), *ndhH*, *rps15*, *ycf1*
Transfer RNA	39	*trnI-CAU* (×2) (i), *trnL-CAA* (×2), *trnV-GAC* (×2), *trnI-GAU* (×2), *trnA-UGC* (×2) (i), *trnR-ACG* (×2), *trnG-GCC* (×2), *trnN-GUU* (×2), *trnH-GUG*, *trnK-UUU* (i), *trnQ-UUG*, *trnS-GCU*, *trnG-UCC*, *trnR-UCU*, *trnC-GCA*, *trnD-GUC*, *trnY-GUA*, *trnE-UUC*, *trnT-GGU*, *trnS-UGA*, *trnG-GCC*, *trnfM-CAU*, *trnS-GGA*, *trnT-UGU*, *trnL-UAA* (i), *trnF-GAA*, *trnV-UAC* (i), *trnM-CAU*, *trnW-CCA*, *trnP-UGG*, *trnL-UAG*
Ribosomal RNA	8	*rrn16* (×2), *rrn23* (×2), *rrn4.5* (×2), *rrn5* (×2)

Phylogenetic analysis (Figure 3) showed that the outgroup (Adoxaceae) was located at the base of the tree, and Caprifoliaceae fell into 7 clades with a highly supported topology of (*Weigela*, (*Lonicera*, ((*Triplostegia*, *Patrinia*), (*Kolkwitzia*, (*Zabelia*, *Morina*))))), which highly supports the molecular taxonomy of Caprifoliaceae (Wang et al. 2020). The *Lonicera* clade is divided into two main clades, namely subgenus *Lonicera* (*L. tragophylla*) and subgenus *Chamaecerasus* (other 24 *Lonicera* species). However, no hierarchical topology was found among or inside sections *Coeloxylosteum* and *Isika. L. caerulea* var. *edulis* (subsection *Caerueae*) clustered with *L. tangutica* (subsection *Purpurascentes*) in the same branch on the clade of section *Isika*, indicating their close phylogenetic relationship. These results challenge the subclassification of the subgenus *Chamaecerasus* (Rehder 1903, Rehder 1913), suggesting that multi-data molecular phylogenetic studies are needed to resolve species relationships and guide taxonomic revisions for the genus *Lonicera*.

## Conclusion

The cp genome characterization and phylogenetic analysis of *L. caerulea* provide useful genetic resources and new insights for future molecular breeding and phylogenetic studies.

## Supplementary Material

Supplemental Material

## Data Availability

The cp assembly in this study is openly available in GenBank of NCBI (https://www.ncbi.nlm.nih.gov/) under accession no. OP345475. The associated BioProject, SRA, and BioSample numbers are PRJNA870700, SRR21133392, and SAMN30383140 respectively.
