# Supplementary material for: Complete chloroplast genome and phylogenetic analysis of Lonicera caerulea var. edulis (Caprifoliaceae)
Source: Mitochondrial DNA B Resour. 2023 Feb 24;8(2):314–8. doi: 10.1080/23802359.2023.2180309 (PMC9970238; doi:10.1080/23802359.2023.2180309)

**Supplementary Materials**

**Supplementary File 1.** Quality control report of the NGS data used for cp assembly


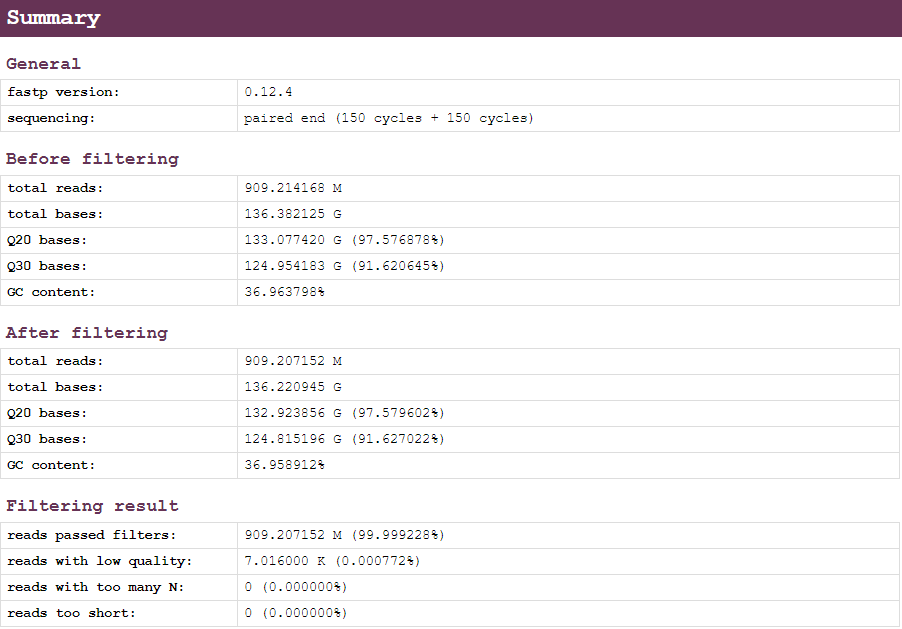

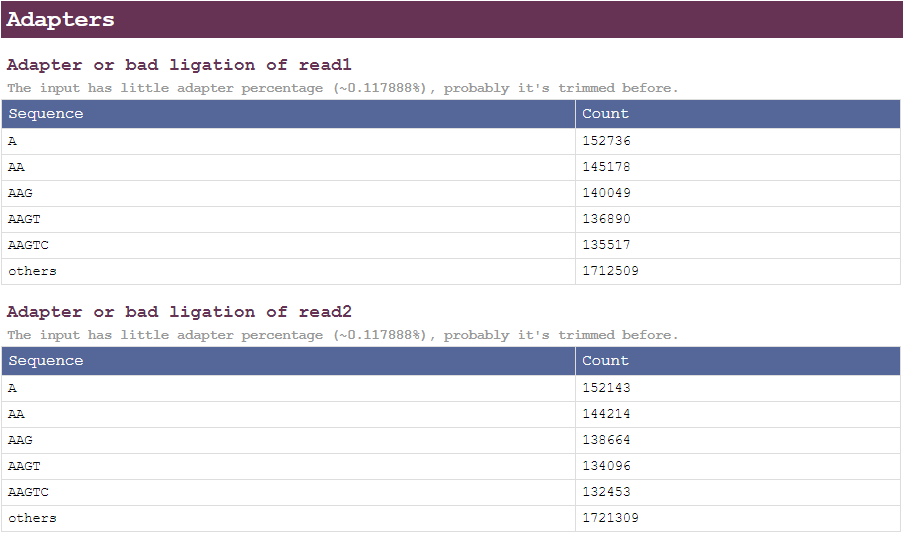

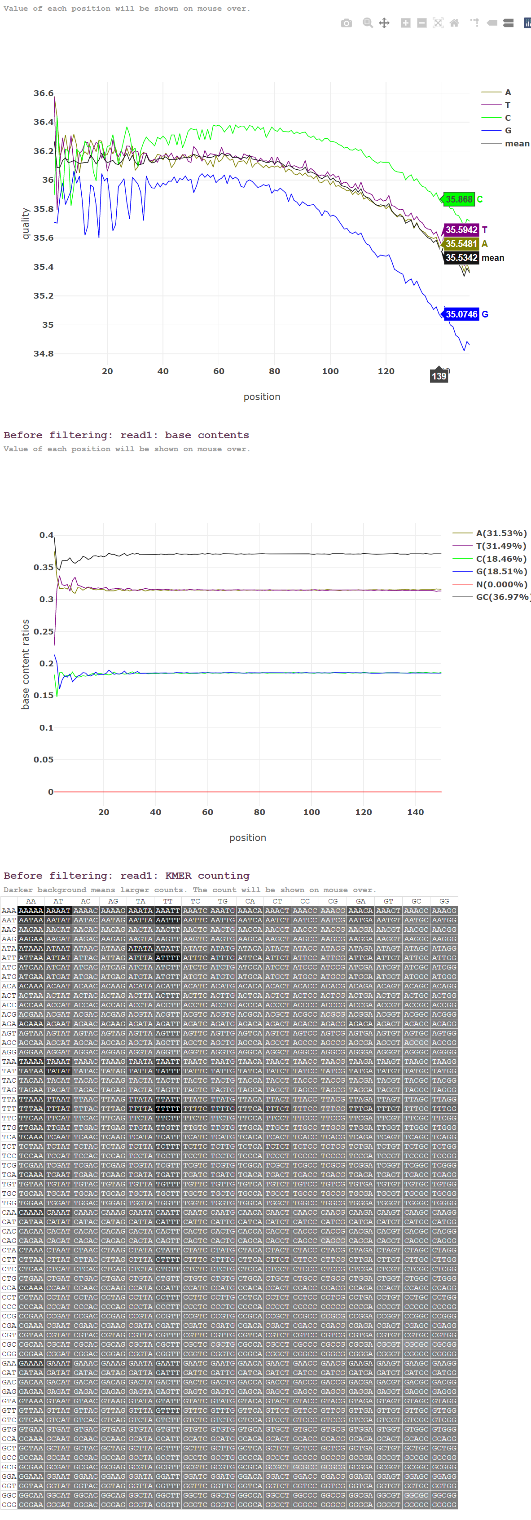

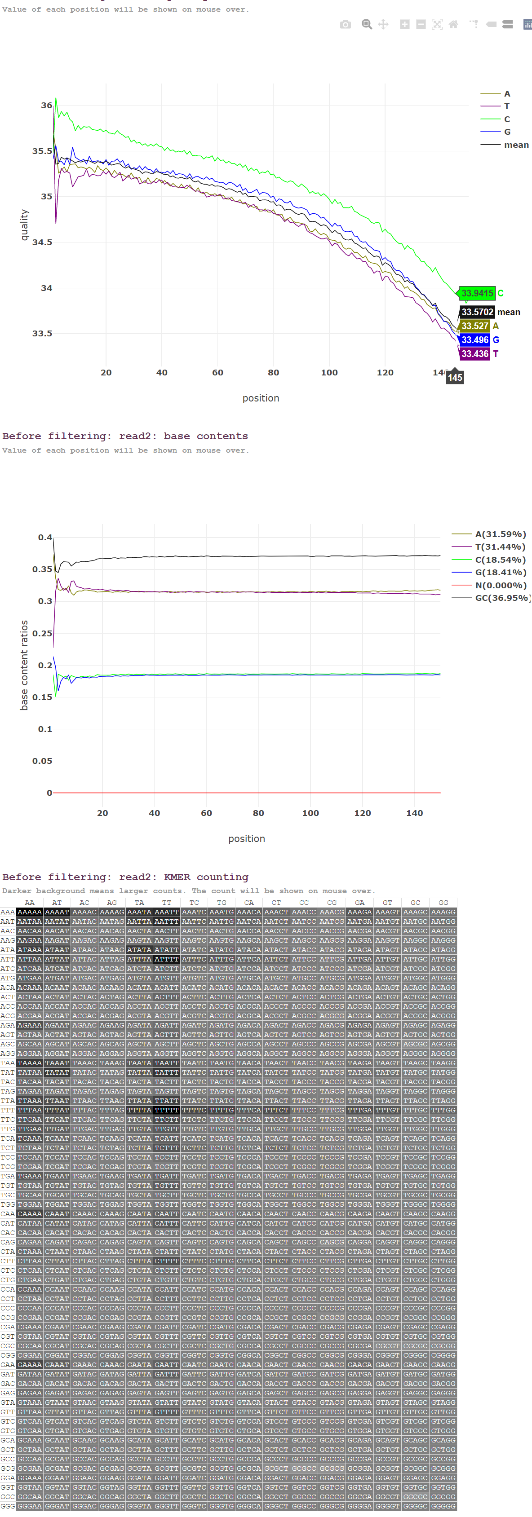


**Supplementary File 2.** Running log file of GetOrganelle


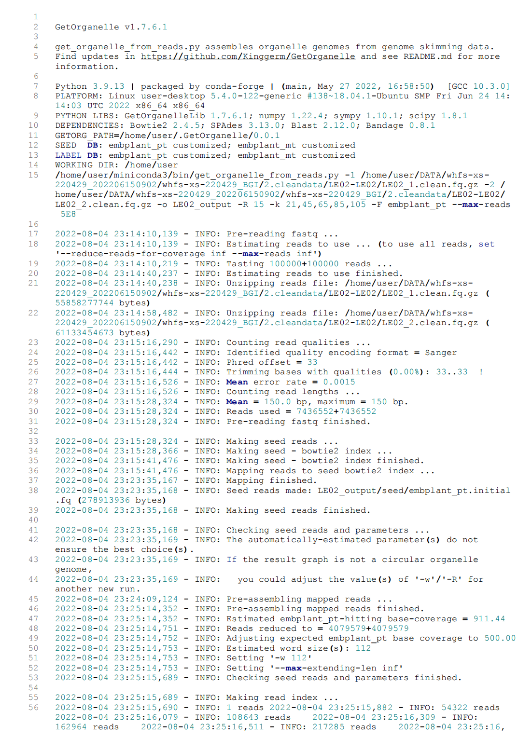

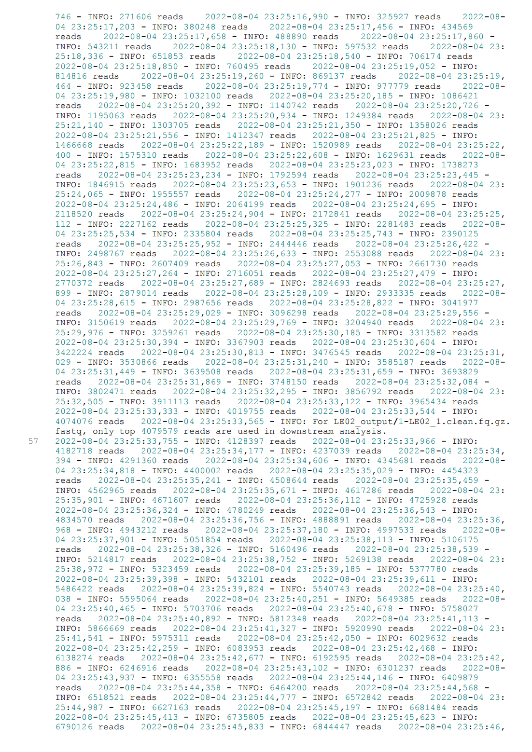

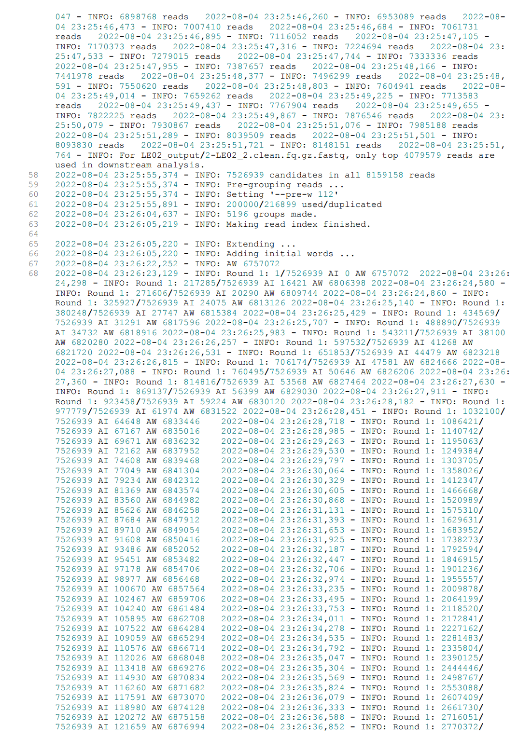

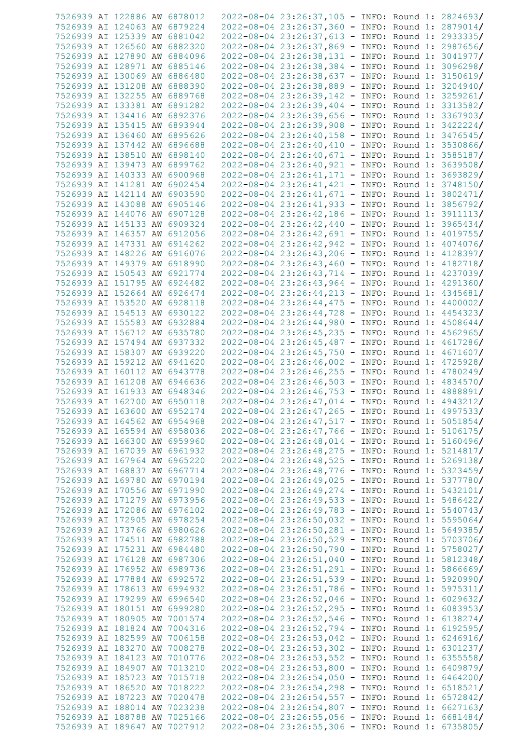

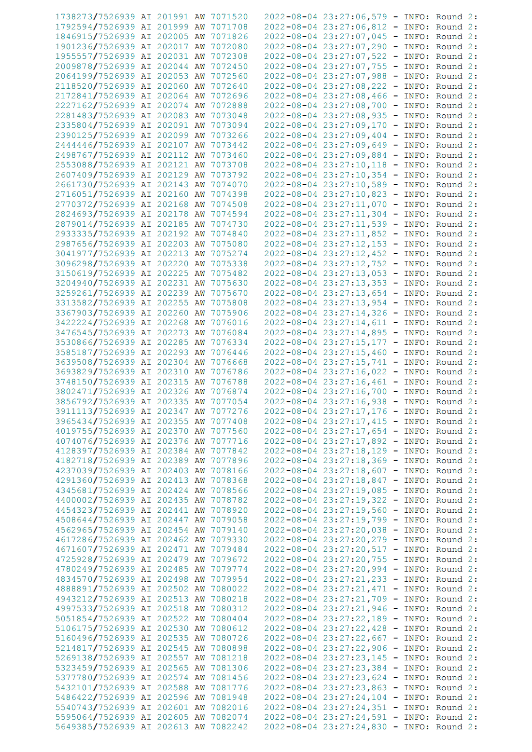

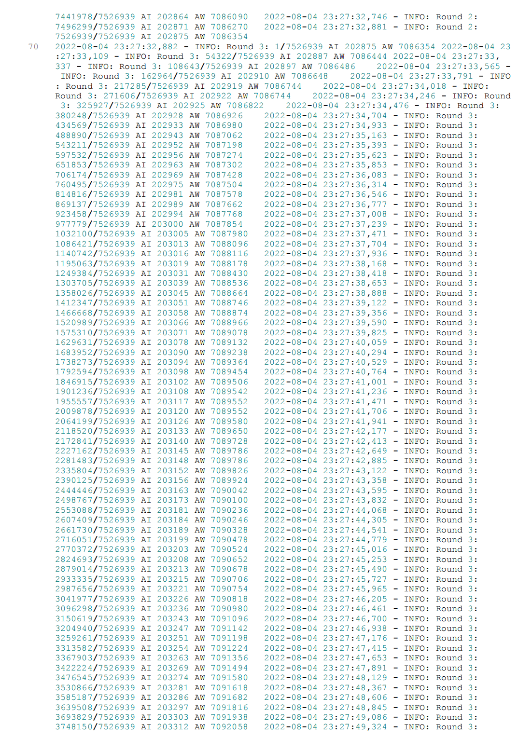

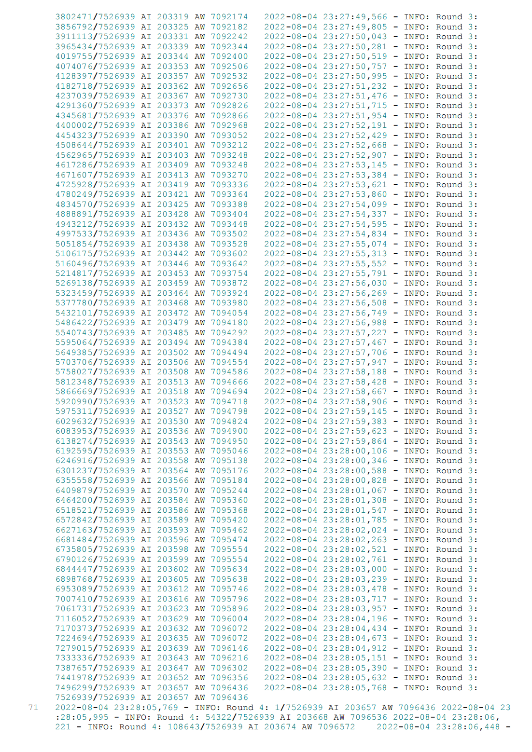

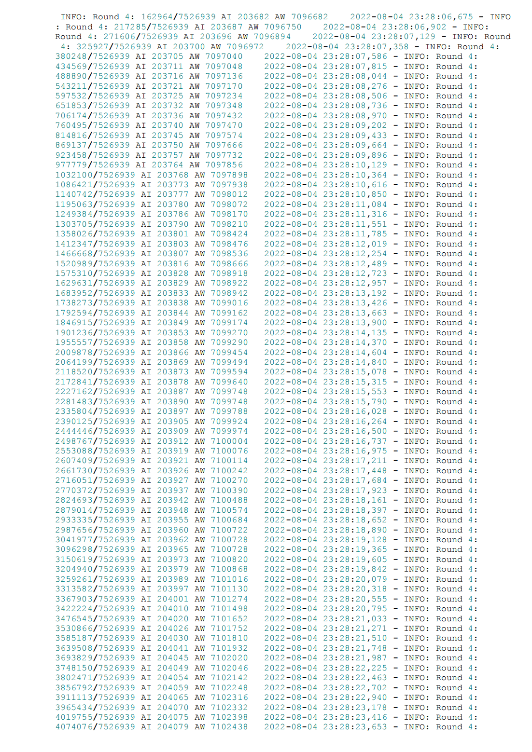

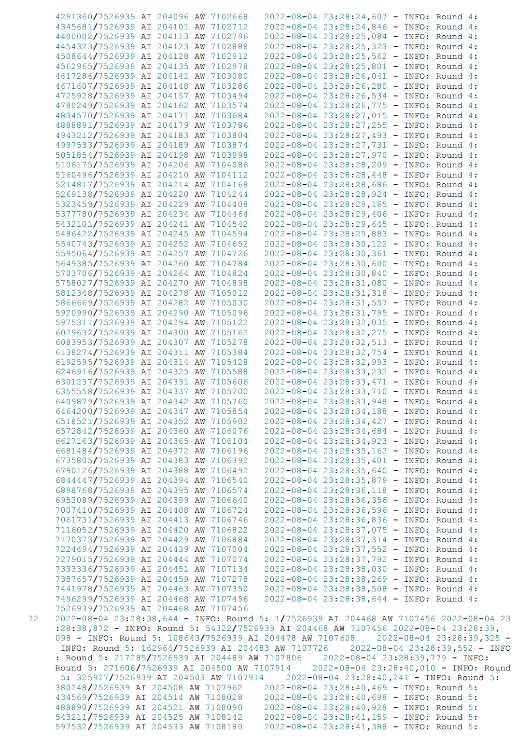

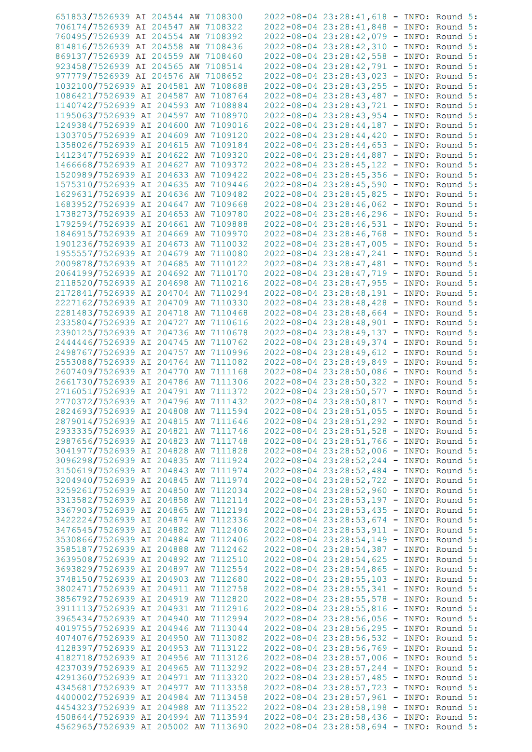

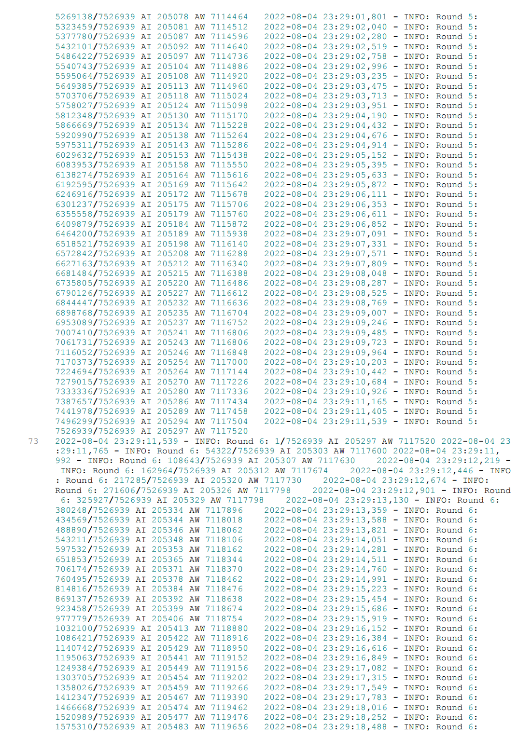

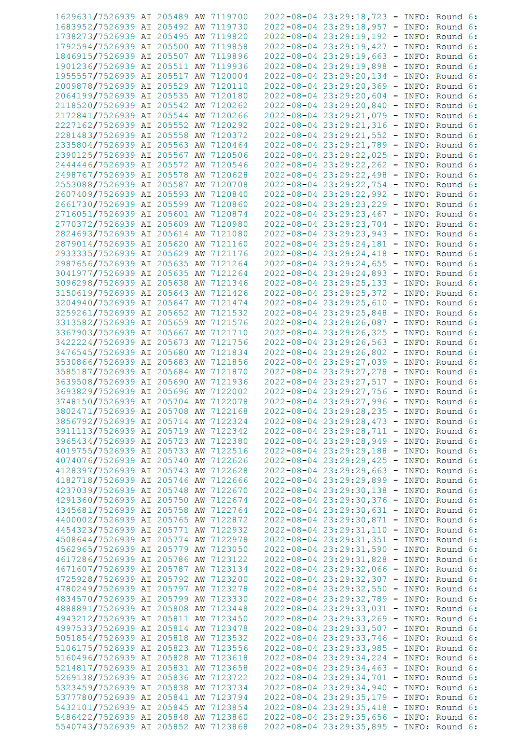

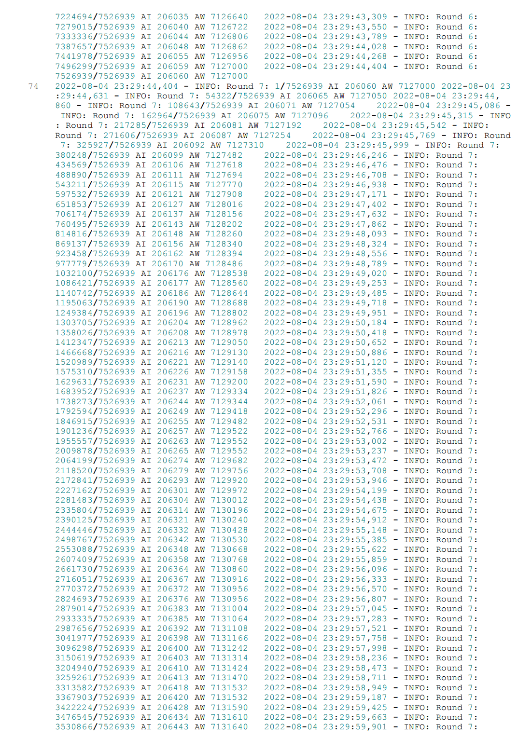

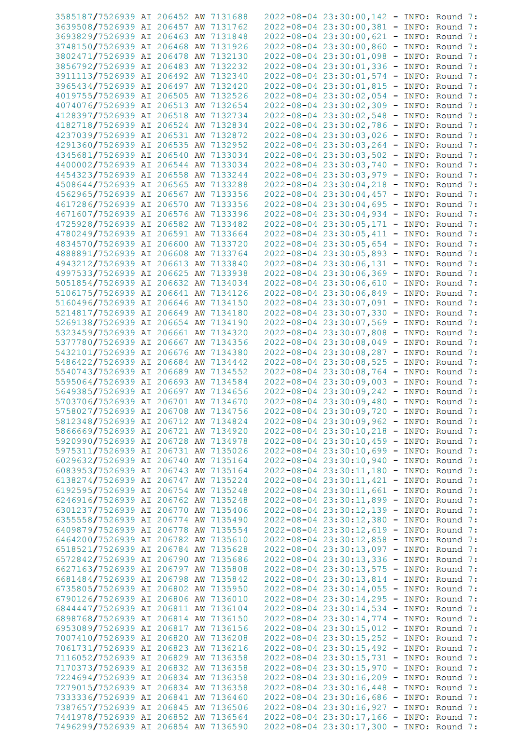

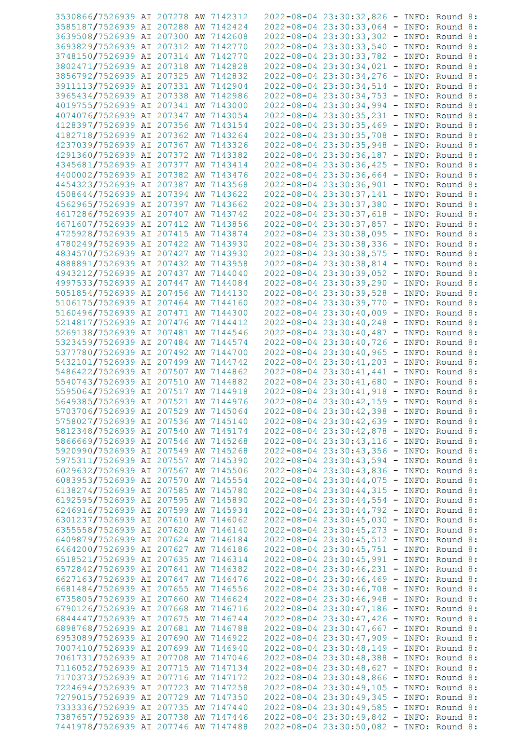

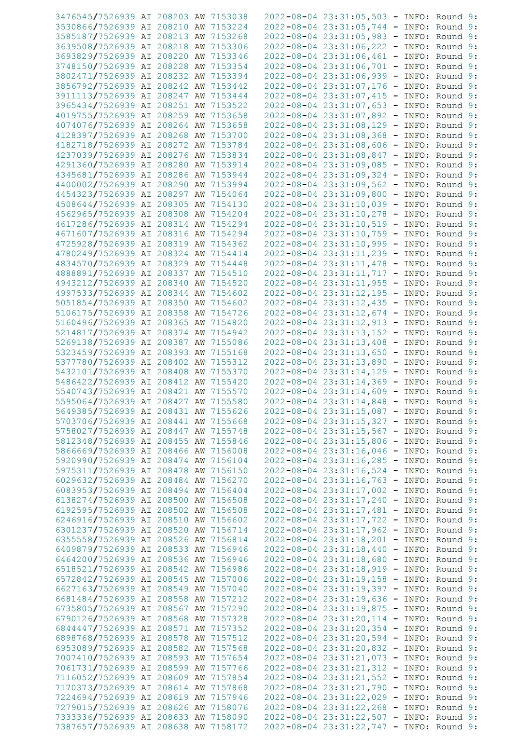

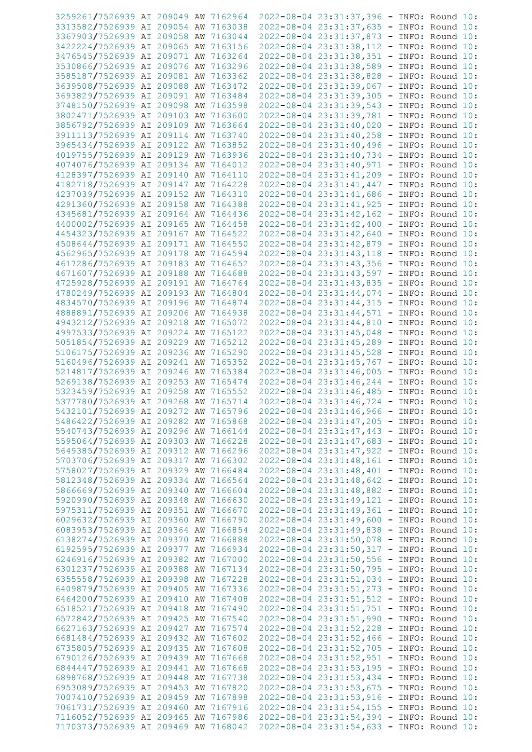

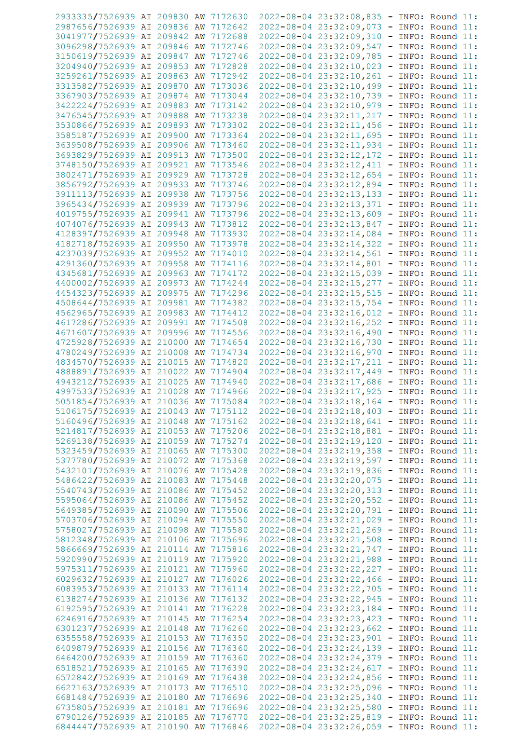

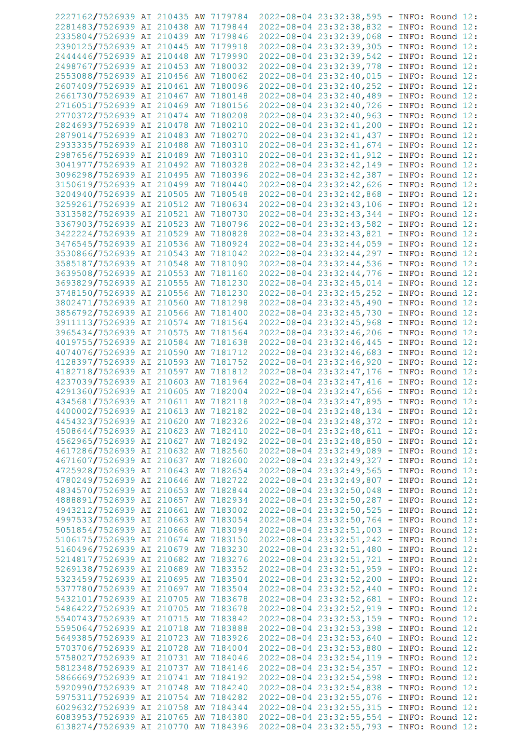

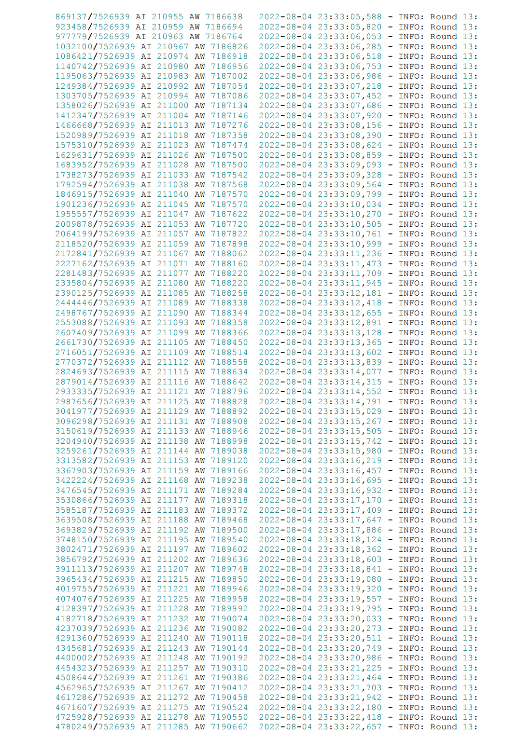

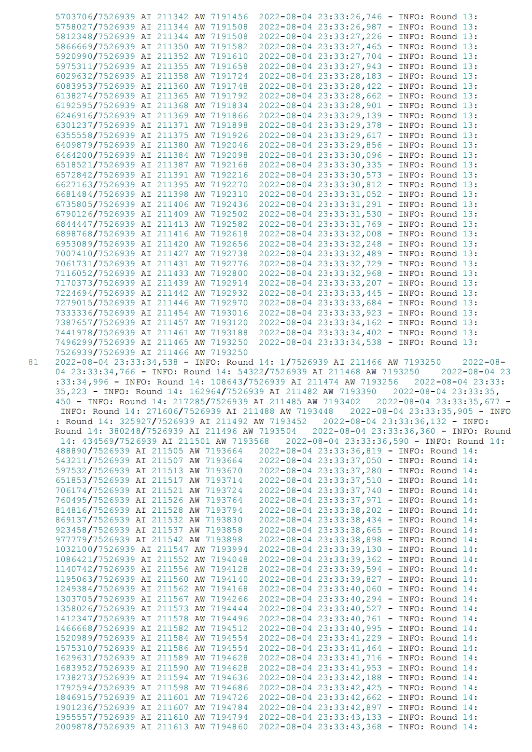

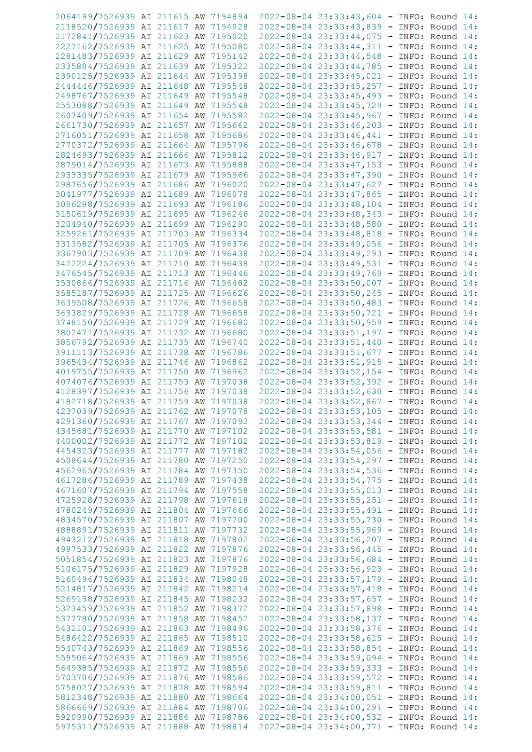

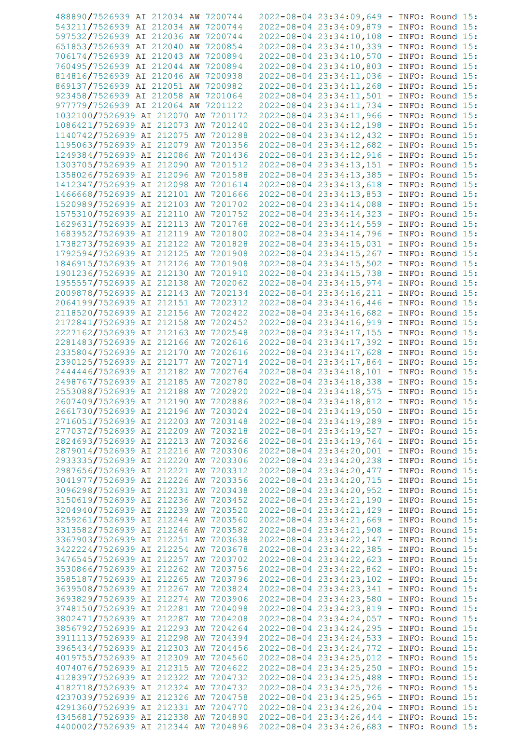

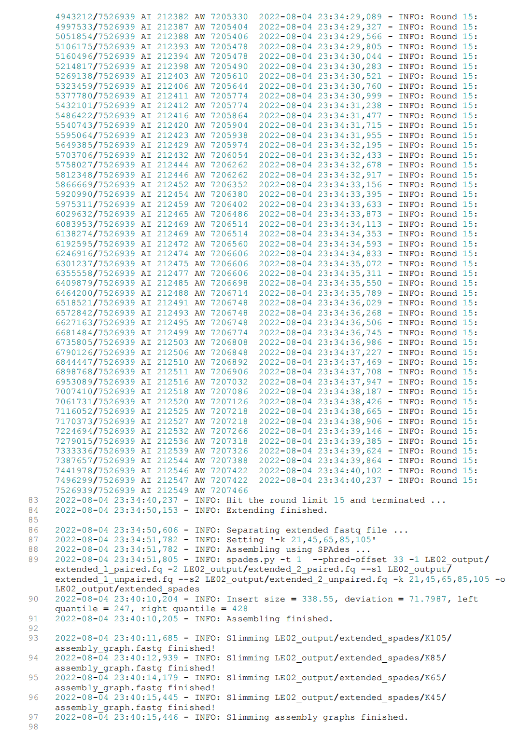

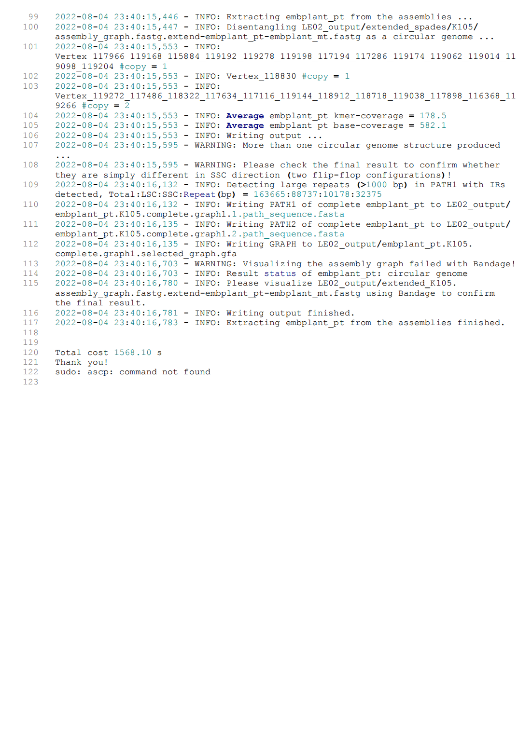


**Supplementary File 3.** Running log of SPAdes (invoked by GetOrganelle)


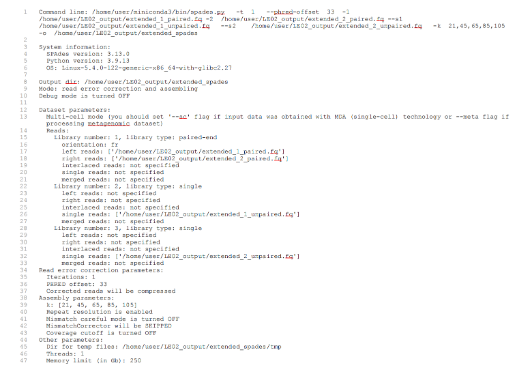

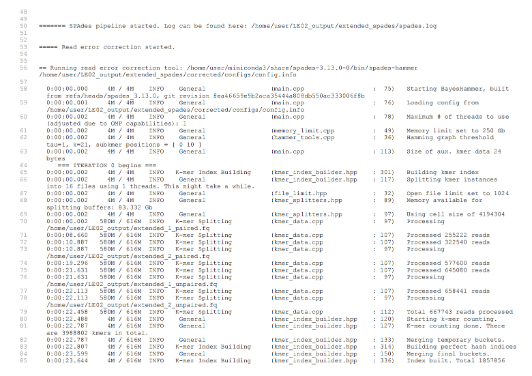

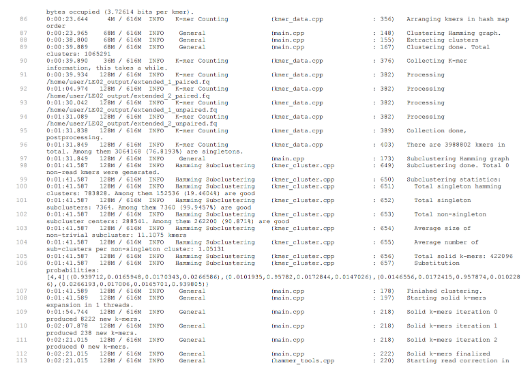

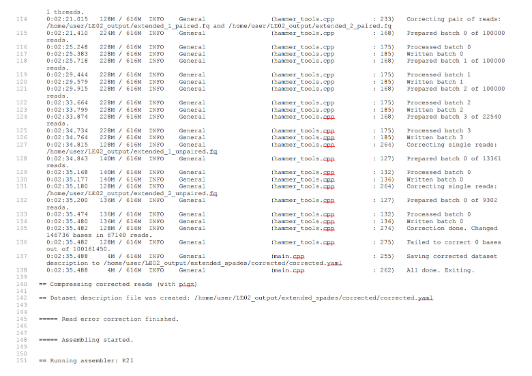

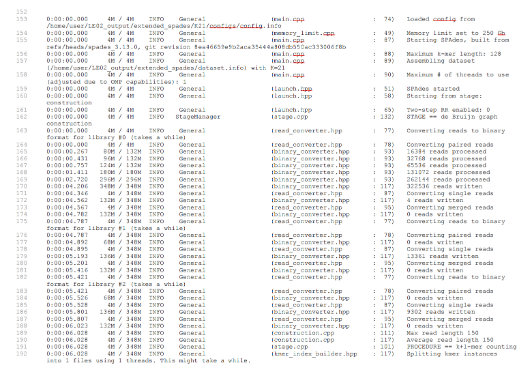

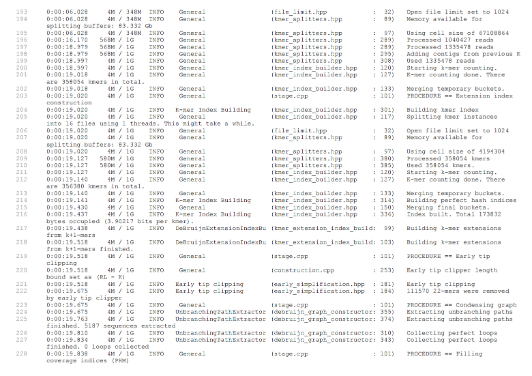

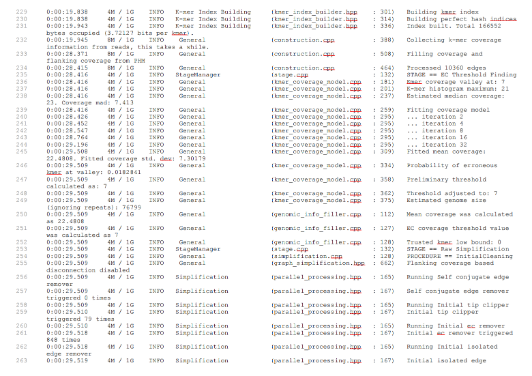

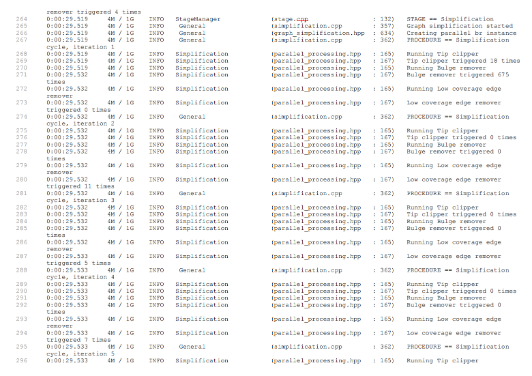

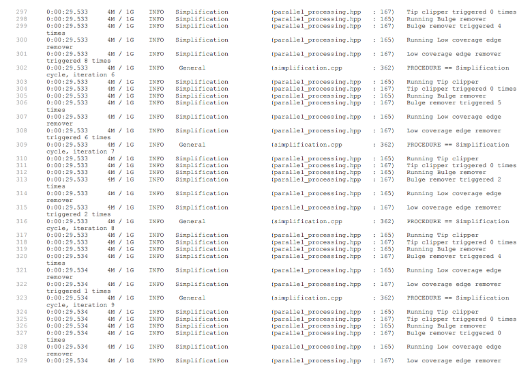

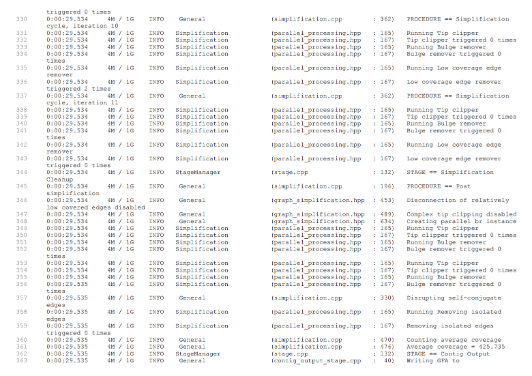

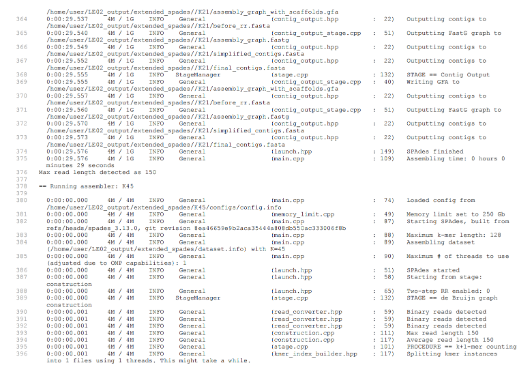

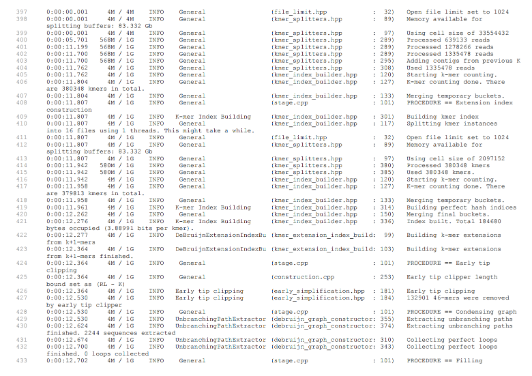

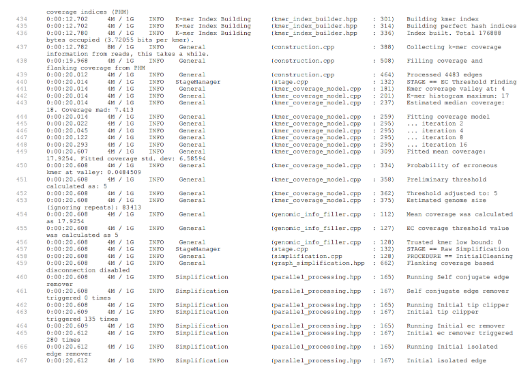

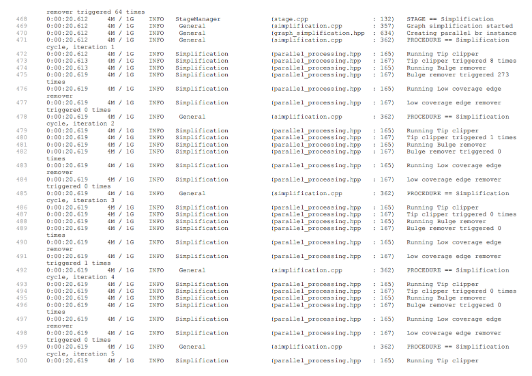

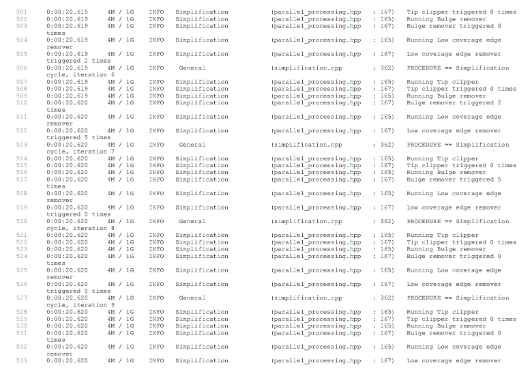

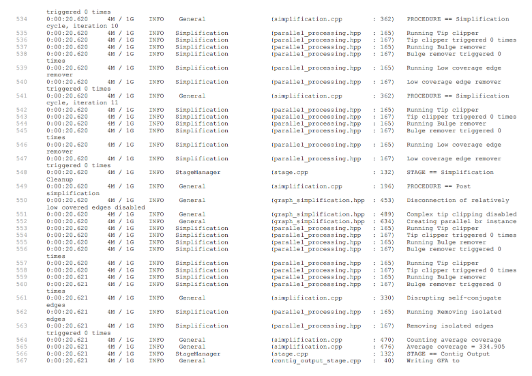

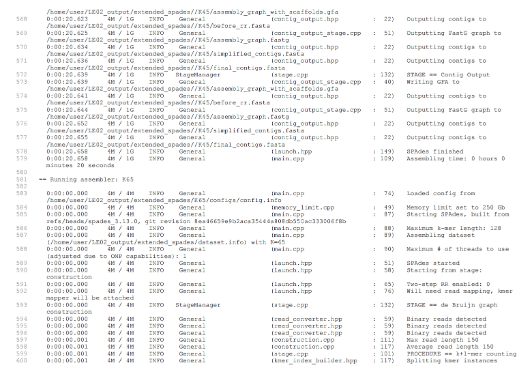

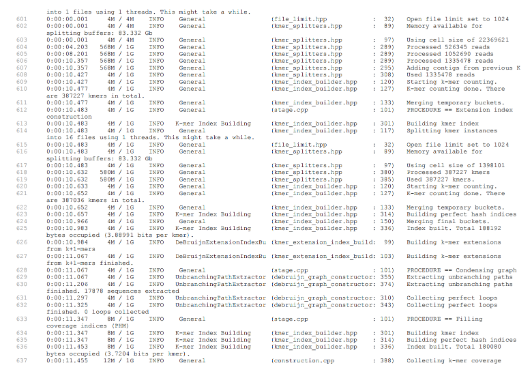

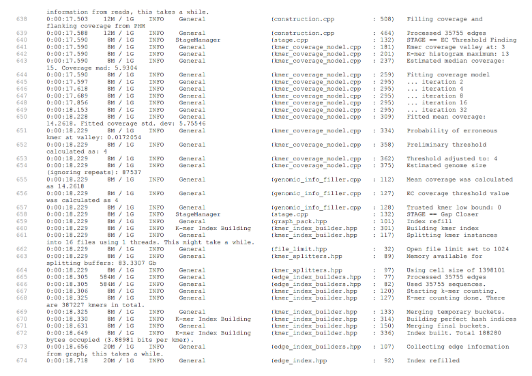

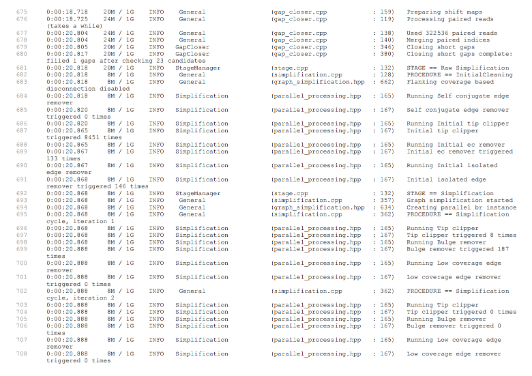

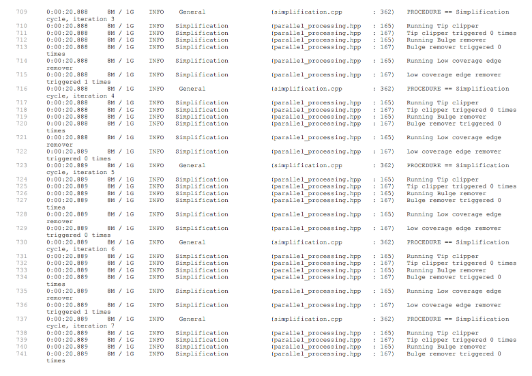

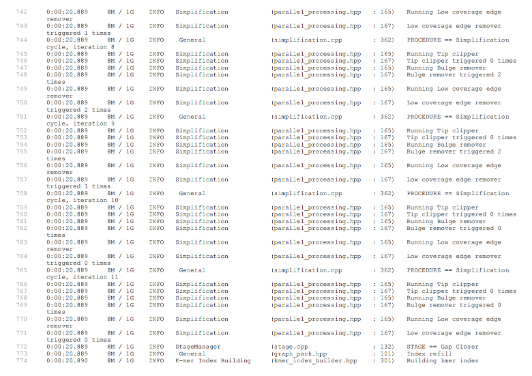

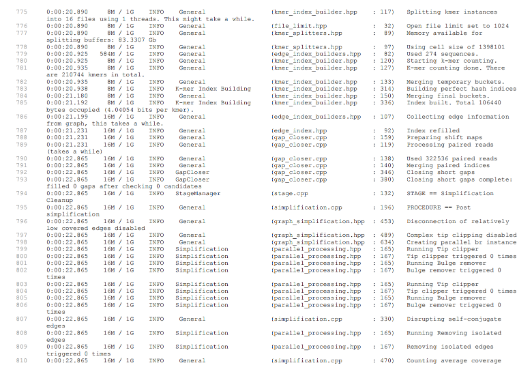

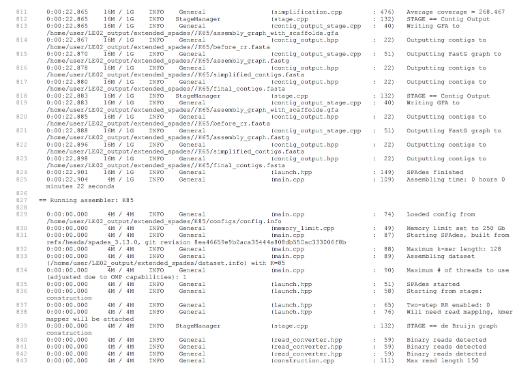

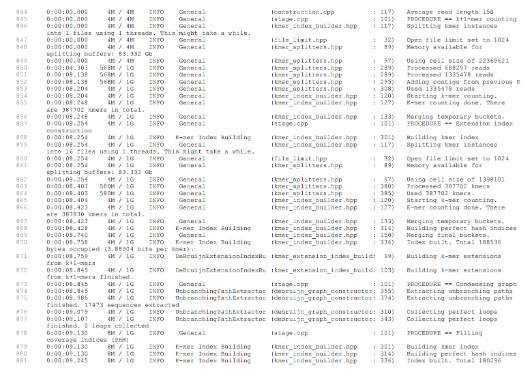

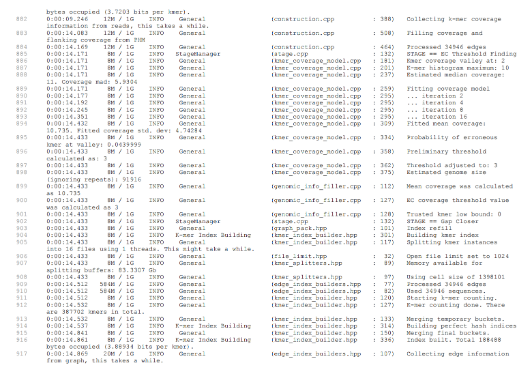

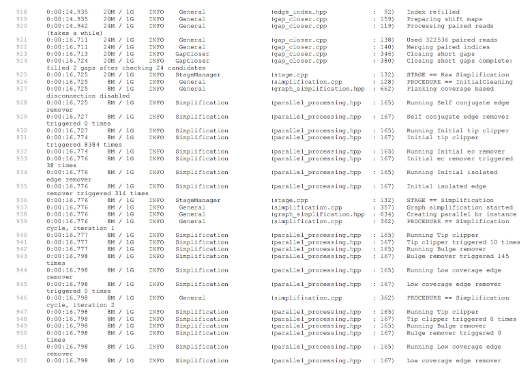

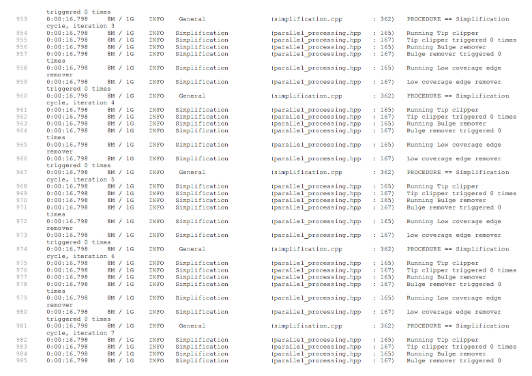

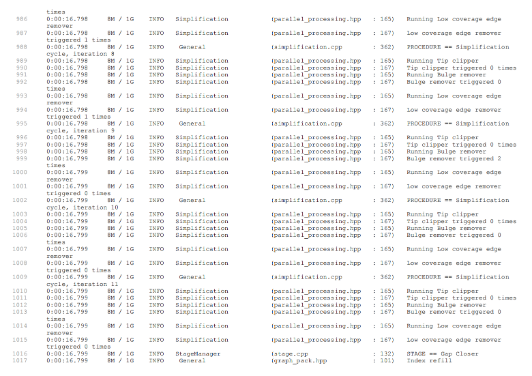

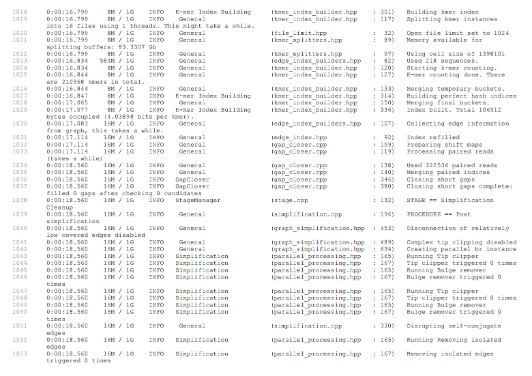

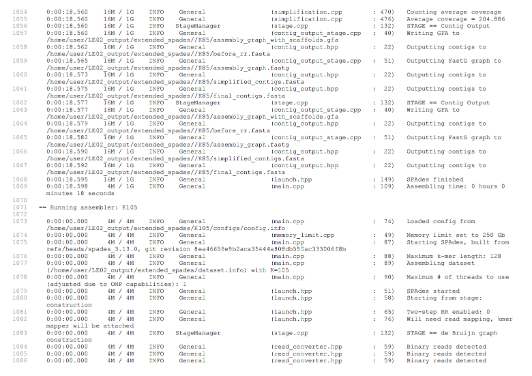

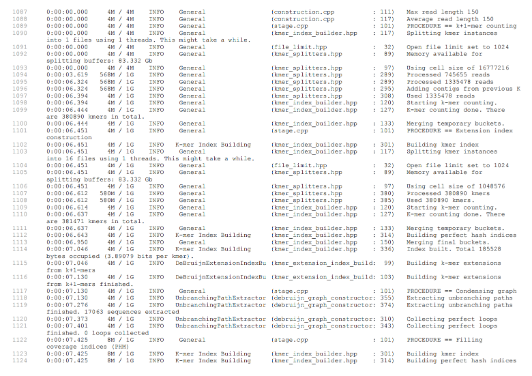

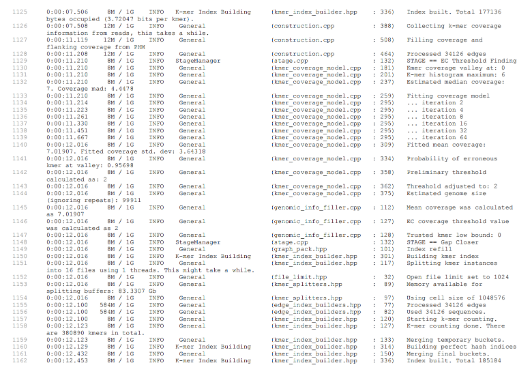

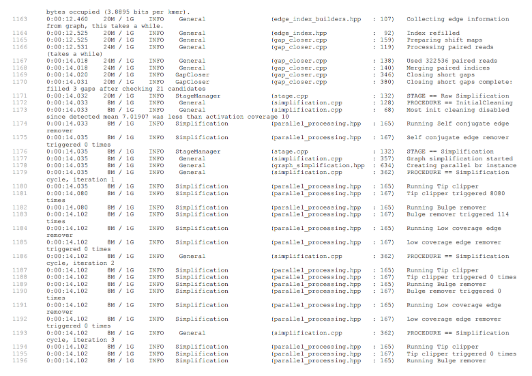

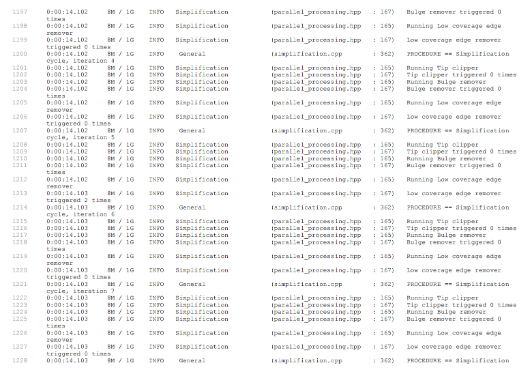

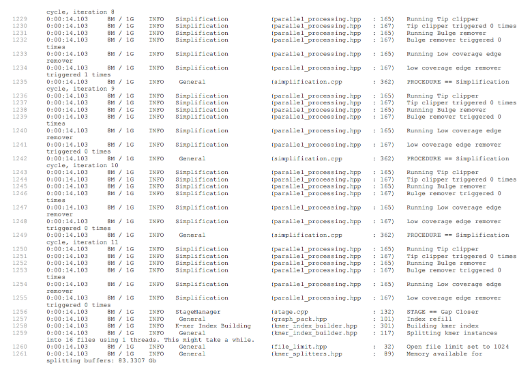

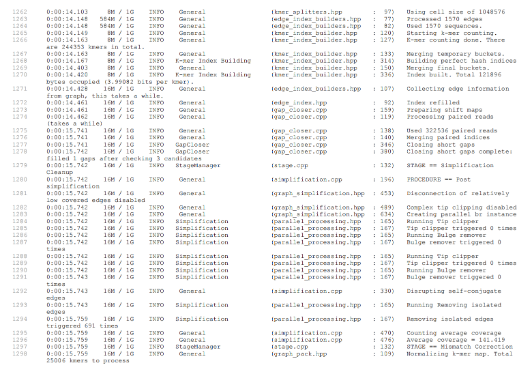

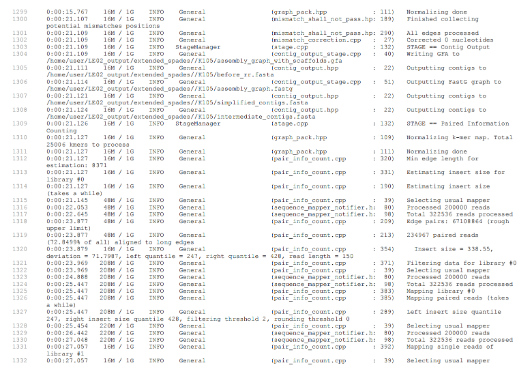

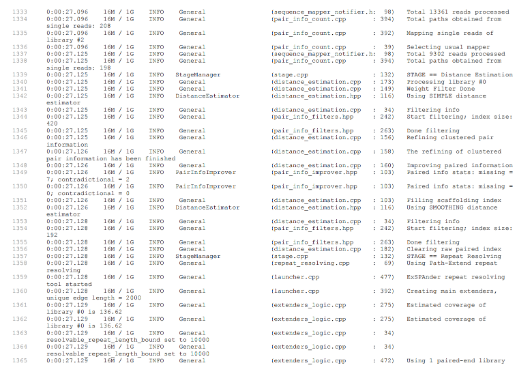

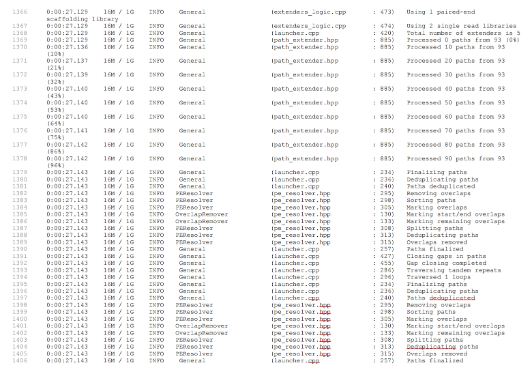

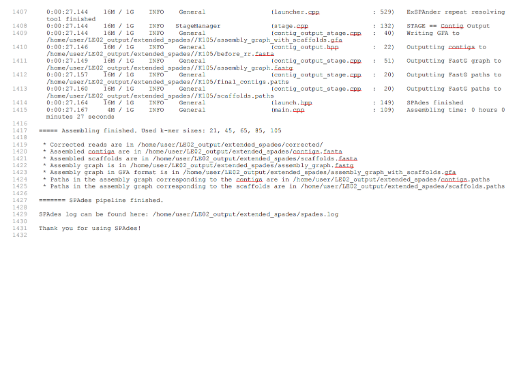


**Figure S1.** Coverage depth figure of the ‘LE02’ cp assembly illustrated by Bandage


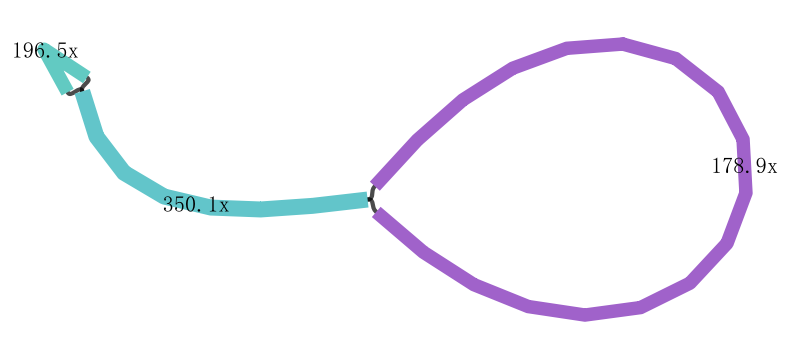


**Figure S2.** Gene structure of the 9 cis−splicing PCGs in the cp genome of *Lonicera caerulea* var. *edulis*


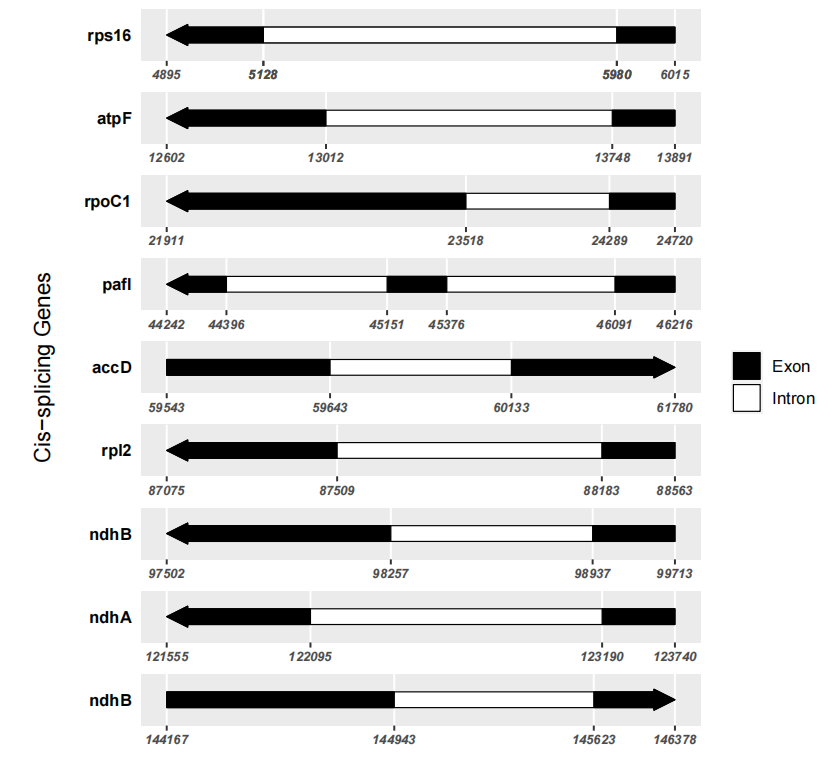


**Figure S3.** Gene structure of the trans−splicing PCG (*rps12*) in the cp genome of *Lonicera caerulea* var. *edulis*


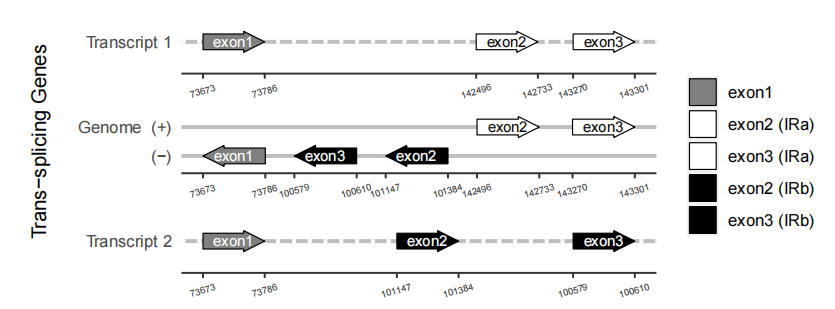

Supplement: Supplemental Material [file TMDN_A_2180309_SM0855.docx]
